# Association Between Intraocular Pressure Changes and Optic Nerve Head and Macular Perfusion Parameters During Isometric Exercise: OCTA Study

**DOI:** 10.3390/diagnostics16030374

**Published:** 2026-01-23

**Authors:** Nina Krobot Čutura, Dominik-Mate Čutura, Maksimilijan Mrak, Ivanka Petric Vicković, Lana Ružić

**Affiliations:** 1 Faculty of Kinesiology, University of Zagreb, Horvacanski zavoj 15, 10 000 Zagreb, Croatialana.ruzic.svegl@kif.unizg.hr (L.R.); 2 Department of Ophthalmology, Varazdin General Hospital, Ivana Mestrovica 1, 42 000 Varazdin, Croatia; 3 Department of Ophthalmology, Karlovac General Hospital, A. Stampara 3, 47 000 Karlovac, Croatia; 4 Department of Ophthalmology, Sestre Milosrdnice University Hospital Centre, Vinogradska cesta 29, 10 000 Zagreb, Croatia

**Keywords:** optical coherence tomography angiography, isometric exercise, retinal perfusion, intraocular pressure

## Abstract

**Background/Objectives:** Optical coherence tomography angiography (OCTA) is a non-invasive imaging method that enables accurate in vivo visualisation and quantification of the macular and optic nerve head microvasculature, providing an indirect assessment of local retinal perfusion. This study aimed to evaluate the changes in OCTA perfusion parameters of macula and optic nerve head in healthy individuals following different isometric exercises and to determine their association with intraocular pressure alterations. **Methods:** Each subject performed four isometric exercises: elbow plank, reverse plank, right-side plank, and wall sit. Measurements of intraocular pressure, systemic blood pressure, heart rate, and OCT angiography of macula and optic nerve head were conducted before each exercise, immediately after its completion, and after a five-minute rest period. Intraocular pressure was measured using a Perkins applanation tonometer, and systemic blood pressure and heart rate were recorded using an automated sphygmomanometer. The relationship between changes in intraocular pressure and OCTA perfusion parameters was analysed. **Results:** A total of 12 eyes of 12 healthy subjects were included in the study, with a mean age of 28.67 ± 2.39 years. An immediate reduction in optic nerve head vessel density was observed after each exercise (elbow plank: *p* = 0.012; wall sit: *p* = 0.009; reverse plank: *p* < 0.001; right-side plank: *p* < 0.001), with a sustained decrease during the rest period following right-side plank. No significant changes in vessel density were observed in the macular region. Heart rate and systemic blood pressure increased after each exercise, while intraocular pressure increased following all exercises except the wall sit. Changes in intraocular pressure were significantly negatively associated with changes in optic nerve head vessel density in the post-rest period following elbow plank (inside disc sector: b = −1.153, *p* = 0.02, peripapillary sector: b = −0.369, *p* = 0.009) and reverse plank (whole image sector: b = −0.589, *p* = 0.031). **Conclusions:** The performance of isometric exercises induced an acute reduction in optic nerve head vessel density, and a significant association with intraocular pressure changes was observed. OCTA represents a promising research tool not only for the assessment of retinal microcirculation but also in the field of sports medicine.

## 1. Introduction

Ocular perfusion is essential for maintaining the structural and functional integrity of the retina. Retinal microcirculation is known to be regulated by intrinsic autoregulatory mechanisms, involving locally released humoral factors, which ensure relatively constant blood flow despite fluctuations in systemic haemodynamic parameters [1]. However, various intrinsic and extrinsic factors may disrupt the retinal structure or vascular function. Dysregulation of ocular vascular responses has been reported in early stages of several vision-threatening conditions, such as glaucoma, diabetic retinopathy, and age-related macular degeneration [2,3,4]. Transient alterations in ocular blood flow have also been observed during everyday activities, including physical exercise [5].

Retinal vascular responses in macula and optic nerve head (ONH) regions to different stimuli can be examined using optical coherence tomography angiography (OCTA). OCTA is a frequently used, non-invasive imaging method that enables in vivo visualisation of the retinal and choroidal microvasculature. This method detects the blood flow-induced change produced by moving blood cells in retinal vessels, allowing an indirect assessment of ocular microvascular perfusion [6]. Its ability to rapidly and accurately assess the macular and optic nerve head microvasculature makes it a valuable diagnostic tool not only in ophthalmology but increasingly in the field of sports medicine as well [7].

Regular physical activity is well known to improve vascular function [8,9], and numerous studies have investigated exercise-induced changes in retinal blood flow using OCTA. The positive impact of high-intensity interval training on retinal perfusion has been demonstrated after the completion of several-week exercise programmes [10,11]. On the other side, studies focusing on short-term effects of dynamic exercise on ONH and macular perfusion observed different results [12,13,14]. For instance, Alnawaiseh et al.’s research on high-intensity dynamic exercise reported a decrease in perfusion of both ONH and macula after the completion of exercises [12]. A reduction in macular blood flow was also observed following 20 min of stationary bicycle exercise in both adult and paediatric populations [13,14].

However, the acute OCTA changes in retinal microvasculature in response to different exercise modalities, such as isometric exercises, have not been extensively studied, and limited research reports conflicting findings. Sousa et al. reported a significant decrease in macular vessel density in both superficial and deep capillary plexus following the handgrip test [15]. In contrast, Brinkmann et al. did not observe any significant acute changes in retinal perfusion after the performance of the handgrip test [16]. Isometric exercises enhance muscle strength and endurance [17] and, unlike dynamic exercise [18], may cause an elevation in intraocular pressure (IOP) [19,20]. Elevated intraocular pressure is known to be the main risk factor for glaucoma and, together with reduced ocular blood flow, has a causative role in the development and progression of disease [21,22].

The aim of the present study was to assess OCTA alterations in optic nerve head and macular perfusion parameters of healthy individuals in response to different isometric exercises and to examine their association with intraocular pressure changes. To our knowledge, this is the first study to investigate the effects of various types of isometric activity, which are widely practised and commonly included in training routines. Also, the examination of the interaction between intraocular pressure and retinal perfusion parameters has not yet been systematically investigated in the context of isometric exercises. The novel aspect of this study was an additional evaluation of retinal vascular responses after a rest period too.

Since blood flow dysregulation has been observed as a risk factor to many ocular diseases [1], it is important to determine the acute effects of different exercise modalities on parameters that may contribute to disease onset or progression.

## 2. Materials and Methods

### 2.1. Participants

The required sample size was calculated using the G*Power programme (version 3.1.9.7., Düsseldorf, Germany). In Alnawaiseh et al.’s research, a change in peripapillary and parafoveal blood flow was found after exercise, with an effect size over 1, meaning that six subjects were required to achieve acceptable statistical power in this study [12]. However, to ensure greater statistical power and to account for potential unpredictable responses, the sample size was increased to twelve.

Five male (41.7%) and seven female (58.3%) subjects with no history of systemic or ocular illnesses were included in this study. The criteria of exclusion were as follows: a refractive error > ±3.00 diopters sphere (DS) and ±1.50 diopters cylinder (DC); baseline intraocular pressure > 21 mmHg; contact lens use; history of ocular surgery or trauma; use of topical or systemic medication within the previous month; subject’s fitness level; locomotor limitations; or inability to perform study-defined exercises. To exclude highly fit individuals, an interview was conducted regarding the implementation of physical exercise. The interview included questions about the frequency of physical exercise in the previous 12 months, reported as the number of days per week, as well as the duration of exercise expressed in minutes per session. The perceived intensity of exercise was assessed and categorised as low, moderate or high. Participants reporting moderate or high-intensity physical activity on three or more days per week, for more than 30 min per session and period longer than 6 months, were excluded from participation. Based on this criteria, two potential subjects were excluded prior to the beginning of the study. The consummation of alcohol, caffeine, or cigarettes was not permitted on the day of the measurement. Prior to participation in the study, all subjects provided informed consent after receiving detailed explanation of the aim, benefit, and risk of the study. The study protocol was approved by the Ethics Committee of Varazdin General Hospital (2186-192-38-24-6, 30 October 2024) and followed the tenets of the Declaration of Helsinki.

### 2.2. Mesaurement Protocol

**Figure 1:**
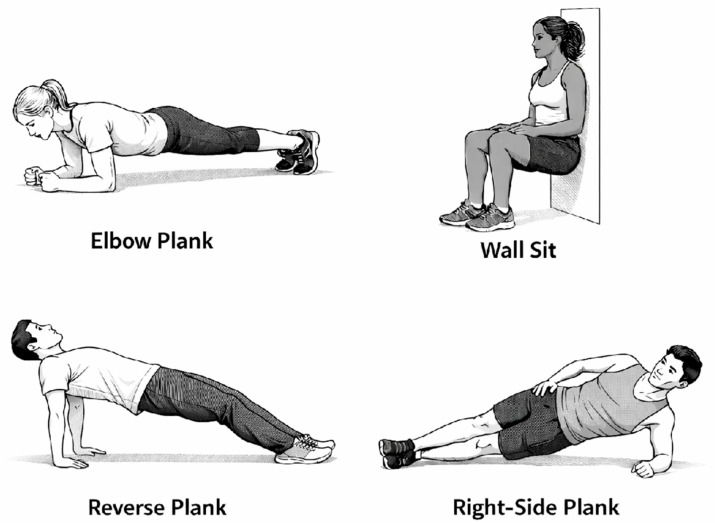
The elbow plank, wall sit, reverse plank, and right-side plank exercises included in the study protocol. The initial figure was generated with the assistance of an AI tool (ChatGPT, OpenAI, GPT-5.2 version) and subsequently modified by the authors.

**Figure 2:**
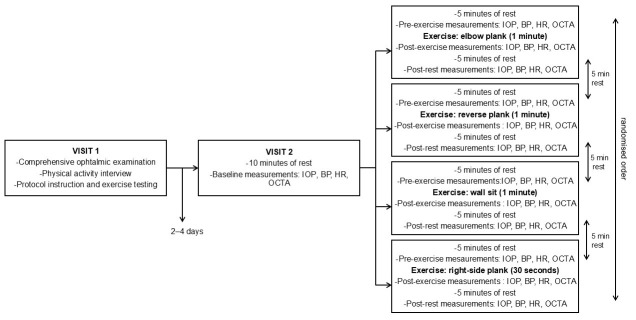
Flow diagram of subjects’ measurement sessions. BP = blood pressure; HR = heart rate; IOP = intraocular pressure; OCTA = optical coherence tomography angiography.

Subjects participated in the testing procedures in two separate visits, scheduled 2 to 4 days apart. In the first visit, a comprehensive ophthalmic examination was performed by an ophthalmologist, including determination of best corrected visual acuity, a slit lamp biomicroscopy, fundoscopy of the macula and optic nerve head, and intraocular pressure measurement using Perkins applanation tonometry. Subsequently, a short interview was conducted to determine participants’ level of physical activity. Participants were then familiarised with the experimental protocol and instructed on how to perform four different isometric exercises: elbow plank, right-side plank, reverse plank, and wall sit (Figure 1). Participants were assessed for eligibility to perform the exercises, and if no exclusion criteria were met, the second visit was scheduled. During the second visit, participants were first asked to rest for 10 min, after which baseline measurements were obtained. The sequence of parameter measurements was fixed: intraocular pressure (IOP) was measured first, followed by systemic blood pressure (BP) and heart rate (HR), and finally OCT angiography of macula and optic nerve head. Subsequently, the experimental protocol was conducted, consisting of four measurement sessions which included four different isometric exercises. Each measurement session followed a structured sequence that was applied consistently across all sessions. At the beginning of each measurement session, participants underwent a five-minute resting period, after which pre-exercise values of parameters were recorded. Participants then performed the exercise, and measurements were obtained immediately after its completion following the same protocol as at baseline. This was followed by a five-minute period of seated rest, after which the same measurements were repeated. Between each measurement session, an additional five-minute rest period was provided for muscular recovery. This additional period of rest also ensured that all measured parameters returned to baseline values prior to the next exercise. The elbow plank, reverse plank, and wall sit were performed for one minute, and the right-side plank was performed for 30 s. The right-side plank was performed for a shorter duration compared to other exercises because of its higher perceived intensity and unilateral load. This adjustment was made to ensure participants complete the exercise with proper form. The order of the measurement sessions was randomised to avoid order bias. The Valsalva manoeuvre was not permitted due to its possible interference with intraocular pressure and retinal vessel density [23,24]. All ocular parameters were measured exclusively on the right eye of each participant, as inter-eye differences were expected to be minimal in healthy participants with no ocular disease. The right eye was chosen for measurements across all participants to ensure methodological consistency, as there was no clinical indication for preference of either eye. This protocol was applied to each of the study-defined exercises (Figure 2).

IOP was measured using a Perkins Mk3 applanation tonometer (Haag-Streit, UK), a portable, hand-held device that is considered the gold standard for rapid and accurate IOP measurements. The topical anaesthetic (tetracaine) and fluorescein were instilled to the eye once at the beginning of each measurement session, ensuring that IOP recording could be performed without an additional waiting period. Three consecutive IOP measurements were taken by an experienced examiner in accordance with standard clinical practice, and the mean value was used for analysis.

BP and HR were measured in the right brachial artery using an automated sphygmomanometer, with the cuff placed at heart level in an upright sitting position. A standardised measurement cycle was applied uniformly across participants and measurement sessions.

OCTA imaging was conducted using the AngioVue Imaging system (RTVue XR Avanti SD-OCT, Optovue, Fremont, CA, USA). Vessel density (VD) was evaluated with integrated Angio Analytics software (version 2017.1.0.155; Optovue, Inc., Fremont, CA, USA). VD is an OCTA parameter that represents the percentage of area occupied by blood vessels with detectable blood flow and is commonly analysed as an indirect indicator of microvascular perfusion status [25]. All measurements were performed exclusively on the right eye, under mesopic lighting conditions, and without prior pupil dilatation. OCTA scans of the optic nerve head and macula were acquired in a 4.5 mm × 4.5 mm and 6.0 mm × 6.0 mm areas. The software automatically generated *en face* retinal angiograms and calculated vessel density in various sectors of the optic nerve head and macula. This included radial peripapillary capillary vessel density (RPC VD) in three sectors of the optic nerve head (the whole image, inside disc, and peripapillary sector), as well as macular VD in the superficial capillary plexus (SCP) of the whole image, fovea, and parafovea. The peripapillary sector was defined as a 1.0 mm annular region extending outside from the optic disc head margin, and the inside optic disc area as a 2.0 mm diameter region of the optic disc head. The whole image area, comprising both the peripapillary and inside optic disc areas, had a diameter of 4.0 mm. The RPC layer was stated between the inner limiting membrane (ILM) and the retinal nerve fibre layer (RNFL). The SCP was segmented from the inner limiting membrane to the inner plexiform layer (IPL). All layer segmentations were performed automatically by the AngioVue Imaging system (RTVue XR Avanti SD-OCT, Optovue, Fremont, CA, USA) . OCTA device was pre-calibrated before each measurement session, ensuring that the imaging was conducted within approximately 60 s after the exercise cessation. All recordings were obtained by the same experienced operator to ensure internal consistency of the imaging data. Each OCTA scan was completed in less than two minutes. A total of 18 out of 144 acquired OCTA scans were excluded due to a signal strength index ≤ 60 or significant motion artefacts and were immediately repeated to ensure data quality.

### 2.3. Data Analysis

Statistical analysis was performed using STATISTICA 14.0 software for Windows. The Shapiro–Wilk test confirmed the normality of the data distribution. Descriptive data were summarised in the form of arithmetic mean and standard deviation. Differences between pre-exercise, post-exercise, and post-rest values of BP, HR, IOP, and macular and RPC VD were analysed using repeated measures analysis of variance (ANOVA). ANOVA was used after inspecting variability plots for their robustness against slight normality violations. Further analysis of statistically significant data was performed by a Fisher LSD post hoc test. The association between the changes in intraocular pressure, systemic haemodynamic parameters, and retinal perfusion parameters was assessed using linear regression analysis. The level of statistical significance for all statistical tests was set at *p* < 0.05.

## 3. Results

A total of 12 eyes of 12 healthy subjects were included in the analysis, with measurements performed exclusively on the right eye of each participant to ensure consistency of data collection. The mean age of participants was 28.67 ± 2.39 years.

### 3.1. Systemic Haemodynamic Parameters

Regarding systemic haemodynamic parameters, systolic blood pressure increased significantly immediately after the completion of each exercise (elbow plank: *p* = 0.002; wall sit: *p* < 0.001; reverse plank: *p* = 0.013; right-side plank: *p* < 0.001), whereas diastolic blood pressure significantly increased after the wall sit (*p* < 0.001) and reverse plank (*p* = 0.043). Heart rate increased following the completion of all exercises (elbow plank: *p* < 0.001; wall sit: *p* < 0.001; reverse plank: *p* < 0.001; right-side plank: *p* < 0.001). The SBP, DBP, and HR values significantly decreased after a 5 min rest period in comparison to post-exercise levels and did not significantly differ from the pre-exercise measurements (Table 1).

### 3.2. Intraocular Pressure

Intraocular pressure increased significantly in the immediate post-exercise period of the elbow plank (*p* < 0.01), reverse plank (*p* < 0.001), and right-side plank (*p* < 0.001). The wall sit exercise did not result in a significant change in IOP (*p* = 0.232). After a 5 min rest period, a significant reduction in IOP was observed when compared to immediate post-exercise values in the elbow plank (*p* < 0.001), reverse plank (*p* < 0.05), and right-side plank (*p* < 0.01). IOP values measured after the 5 min rest period did not differ significantly from pre-exercise values in any of the exercises (elbow plank: *p* = 0.146; reverse plank: *p* = 0.157; right-side plank: *p* = 0.212) (Table 1).

**Table 1:** Systemic variables and intraocular pressure responses to four isometric exercises.

		Pre-Exercise	Post-Exercise	Post-Rest	*p*-Value *
Elbow plank	SBP (mmHg)	121.67 ± 7.25	129.08 ± 8.55	116.42 ± 6.95	**0.00002**
	DBP (mmHg)	77.17 ± 6.25	75.75 ± 4.71	75.75 ± 3.86	0.564
	HR (bpm)	72.83 ± 8.71	85.33 ± 9.71	75.58 ± 6.37	**0.00001**
	IOP (mmHg)	12.58 ± 3.06	14.58 ± 2.27	11.75 ± 2.90	**0.00013**
Wall sit	SBP (mmHg)	120 ± 5.59	134.58 ± 13.27	121 ± 9.51	**0.00053**
	DBP (mmHg)	78.33 ± 7.77	72.08 ± 5.32	75.17 ± 5.97	**0.00077**
	HR (bpm)	72.58 ± 8.68	86.08 ± 11.15	73.42 ± 9.68	**0.00001**
	IOP (mmHg)	13.25 ± 2.34	13.42 ± 2.23	12.42 ± 2.23	0.232
Reverse plank	SBP (mmHg)	118.25 ± 7.91	122.17 ± 7.91	115.83 ± 6.79	**0.00094**
	DBP (mmHg)	79.17 ± 5.98	74.50 ± 5.21	77.08 ± 8.92	**0.04263**
	HR (bpm)	73 ± 5.10	84.25 ± 11.09	73.67 ± 8.25	**0.00002**
	IOP (mmHg)	12.50 ± 2.50	14.33 ± 2.42	13.17 ± 2.59	**0.00204**
Right-side plank	SBP (mmHg)	116.42 ± 6.96	124.58 ± 6.23	119.25 ± 9.72	**0.00241**
	DBP(mmHg)	75.25 ± 3.96	73.42 ± 4.06	76.25 ± 5.45	0.122
	HR (bpm)	74.67 ± 5.63	86.58 ± 8.54	73.92 ± 5.53	**0.00000**
	IOP(mmHg)	11.92 ± 2.07	14.50 ± 2.91	12.67 ± 2.50	**0.00067**

Values are given as mean ± SD. * Differences between three time points: pre-exercise, post-exercise, and post-rest (after 5 min of rest) were tested using the repeated measure ANOVA. Bolded *p*-values indicate statistically significant differences. SBP = systolic blood pressure, DBP = diastolic blood pressure, HR = heart rate, IOP = intraocular pressure.

### 3.3. Optic Nerve Head and Macular Vessel Density

**Figure 3:**
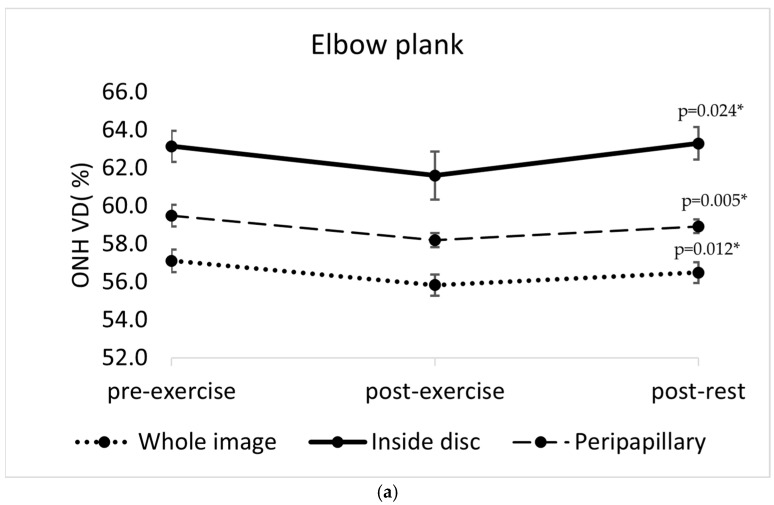

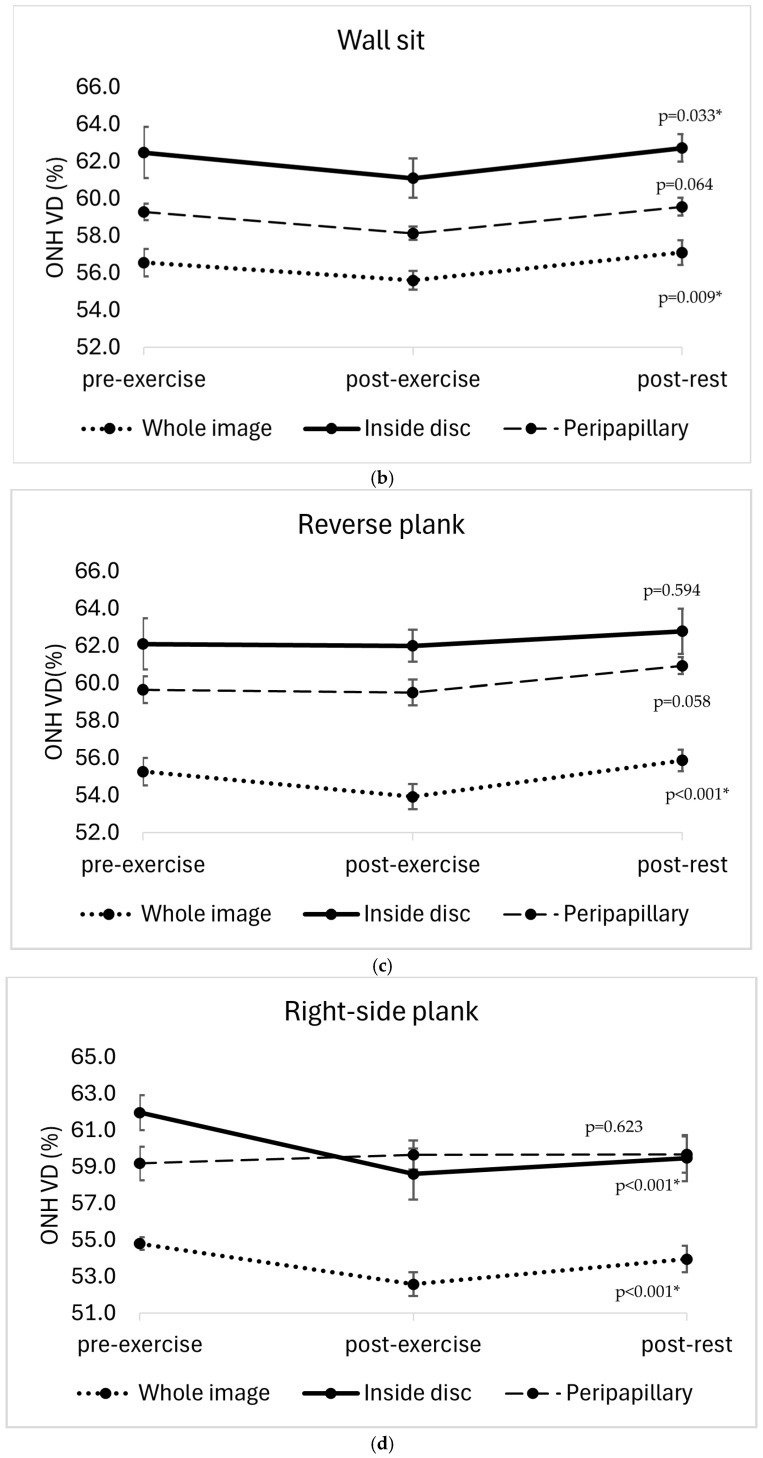
Changes in optic nerve head vessel density (ONH VD, %) across three time points (pre-exercise, post-exercise, and post-rest) in different sectors of the optic nerve head (whole image sector, inside disc sector, peripapillary sector) in four isometric exercises: (**a**) elbow plank; (**b**) wall sit; (**c**) reverse plank; (**d**) right-side plank. The error bars represent the standard error of the mean. * *p* < 0.05 indicates statistically significant results.

A statistically significant reduction in optic nerve head vessel density (ONH VD) was observed immediately after the completion of all four isometric exercises. The changes were significant for the whole image sector in each exercise (elbow plank: F(2, 22) = 5.41, *p* = 0.012; wall sit: F(2, 22) = 5.961, *p* = 0.009, reverse plank: F(2, 22) = 11.771, *p* < 0.001; right-side plank: F(2, 22) = 13.26, *p* < 0.001). After the elbow plank, a decrease in ONH VD was also significant in the peripapillary sector (*p* = 0.05) and inside disc sector (*p* = 0.024). A ONH VD reduction was also significant in the inside disc sector after the wall sit (*p* = 0.033) and right-side plank (*p* < 0.001). After the right-side plank, the reduction in ONH VD persisted after five minutes of rest (the whole image sector: *p* = 0.004; inside disc sector: *p* = 0.001), whereas in the other three exercises, the values returned close to baseline after the post-rest period (Figure 3).

No significant changes in macular vessel density were observed in response to any of the exercises performed (the elbow plank: F(2, 22) = 0.17, *p* = 0.845; wall sit: F(2, 22) = 0.001, *p* = 0.999; reverse plank: F(2, 22) = 0.164, *p* = 0.849; right-side plank: F(2, 22) = 0.299, *p* = 0.745).

### 3.4. Association Between Changes in IOP, ONH VD, and Systemic Haemodynamic Parameters

There was a significant negative association between changes in IOP and changes in ONH vessel density after a 5 min rest period in the elbow plank (inside disc sector: b = −1.153, *p* = 0.02, peripapillary sector: b = −0.369, *p* = 0.009) and reverse plank (whole image sector: b = −0.589, *p* = 0.031) (Table 2). The changes were not significantly associated in acute post-exercise measurement.

An association between ΔHR, ΔSBP, ΔDBP, and ΔONH VD parameters was not observed in any exercise.

## 4. Discussion

This study aimed to evaluate the acute response of ONH and macular perfusion parameters, assessed by OCT angiography to various isometric exercises, and their association with changes in intraocular pressure. The primary finding was an immediate decrease in ONH vessel density (ONH VD) as a response to all four exercises. Reduced ONH VD may reflect a decrease in detectable microvascular perfusion. Our finding is difficult to compare to previous studies, as to the best of our knowledge, this is the first OCTA study to examine changes in ONH VD in the context of isometric exercises. A reduction in ONH VD after exercise has previously been reported in Alnawaiseh et al.’s study but in the setting of dynamic exercises [12].

In the post-exercise period, ONH VD returned close to baseline values within five minutes for all exercises, except the right-side plank. This study demonstrates for the first time that ONH VD can remain significantly reduced even in the post-exercise period following isometric exercise. Notably, the prolonged reduction in ONH VD was observed only after the right-side plank and not after the other isometric exercises included in the study. This finding may suggest that this is an exercise-specific response rather than a general effect of isometric contraction. The right-side plank may require greater muscle engagement and stabilisation in comparison to the other three exercises. Although it was performed for a shorter duration, its higher technical complexity due to unilateral load may contribute to the sustained reduction in ONH vessel density observed post-exercise. However, this explanation remains hypothetical and should be confirmed in future studies.

The observed temporary reduction in ONH VD may be of greater clinical relevance in individuals with pre-existing ocular vascular disorders, as their retinal microcirculation may already be compromised. However, our findings are limited to healthy participants and cannot be extrapolated to other populations. Also, vessel density represents an indirect indicator of perfusion, as it does not quantify blood flow volume or velocity. Given that in patients with ocular vascular disorders even transient alterations in microvascular perfusion may contribute to disease progression; further studies are needed in these populations.

In the present study, the observed vessel density reduction was limited to the ONH region only, with no significant changes in the macular area due to study-defined isometric exercises. Recent OCTA studies investigating the impact of isometric exercises on macular perfusion reported conflicting results. Our finding is in accordance with Brinkmann et al.’s research, which demonstrated the efficiency of autoregulation mechanisms in maintaining stable macular perfusion after isometric exercise [16]. In contrast, Sousa et al. reported the vasoconstrictive response of retinal vasculature to isometric exercise and the consequential decrease in macular vessel density [15].

The difference between macular and ONH vascular response in this study may suggest that the ONH region is more susceptible to transient elevations in IOP, which were observed after each exercise, except for the wall sit. The probable explanation is in greater sensitivity of lamina cribrosa to a mechanical compression from elevated IOP, which is a contributor to ONH damage in glaucoma [26]. The increase in IOP after isometric exercises has been previously reported, although findings on this topic remain inconsistent [19,20,27,28,29].

A significant negative association was found between the change in IOP and change in ONH vessel density in the post-rest period of the elbow plank and reverse plank. This suggests that a reduction in IOP may contribute to the restoration of detectable local microvascular perfusion. After the right-side plank, the alteration in IOP was not significantly associated with ONH VD change, which may be expected considering that VD remained reduced even after the IOP returned to baseline.

Furthermore, the finding that ONH VD was altered after wall sit exercise, despite the absence of significant IOP elevation, also demonstrates the complexity of ocular perfusion regulation beyond IOP alone.

Interestingly, in the acute post-exercise period, there was no association between the increase in IOP and decrease in ONH VD. This may also imply that other physiological mechanisms modulate perfusion of the optic nerve head independently of IOP.

A strong interaction between the IOP and ocular perfusion has been reported before, but only few studies have examined this relationship in the context of physical exercise. For example, a decrease in macular and peripapillary vessel density was demonstrated to correlate significantly with IOP elevation [30,31,32]. Specifically, in the peripapillary region, an IOP increase of 5 to 10 mmHg was associated with a significant reduction in vessel density as measured by OCTA [32]. However, in these studies the IOP spikes were experimentally induced with laser peripheral iridoplasty (LPI), without alteration of the systemic haemodynamic parameters. To compare, our study demonstrated a significant, but not so excessive, acute elevation in intraocular pressure (mean ± SD: 1.65 ± 2.04 mmHg; range: −5.00 to +6.00 mmHg), and it was not observed in all exercises (e.g., wall sit). Nevertheless, the IOP increase was still associated with reduced ONH vessel density in some exercises, but, in contrary to the studies mentioned, systemic haemodynamic parameters were also altered.

Performing isometric exercises in our study elicited an expected acute elevation of systolic blood pressure and heart rate [15,16], but their association with changes in ONH vessel density has not been observed. In contrast, some studies have demonstrated a negative correlation between systolic blood pressure and vessel density [12,13]. However, different types of exercise were examined and IOP measurements were not obtained, which makes a direct comparison difficult. One possible explanation is that the magnitude and duration of SBP elevation in our study was insufficient to disrupt retinal autoregulation, due to the short duration of exercises.

This study has several limitations that should be acknowledged. First, a small sample size limits the ability to generalise the findings to a broader population. Second, only healthy subjects without ocular and systemic diseases were included in the study, so it remains unclear whether individuals with pre-existing ocular vascular disorders would exhibit different responses. Finally, the exercise intensity was not individually adjusted nor systematically quantified; therefore, inter-individual differences in muscle strength may have contributed to variability in the observed ocular responses.

Future research on retinal vascular responses to common physical exercise is warranted for better basic understanding of the ONH blood flow, as it may contribute to strategies for the prevention and treatment of ischaemic disorders of the ONH. Such knowledge would also support the clinical evaluation of novel therapeutic strategies based on the improvement of ocular perfusion, especially in conditions such as glaucoma and vascular ocular disorders.

## 5. Conclusions

In conclusion, this study demonstrated that isometric exercises, including the elbow plank, reverse plank, right-side plank, and wall sit, can lead to an acute reduction in optic nerve head perfusion parameters, as measured by OCT angiography. A sustained reduction in optic nerve head vessel density may occur following the right-side plank. The negative association between changes in intraocular pressure and optic nerve head vessel density found after the elbow plank and reverse plank, and not after the wall sit and right-side plank, demonstrates the complexity of ocular perfusion regulation. Optical coherence tomography angiography proves to be a valuable tool for the evaluation of the retinal vascular system but also a potential instrument in sports medicine research.

## Figures and Tables

**Table 2:** Linear regression analysis of association between intraocular pressure changes and ONH vessel density changes across different ONH sectors (whole image sector, inside disc sector, peripapillary sector) after the rest period.

	Whole Image (b/*p*)	Inside Disc (b/*p*)	Peripapillary (b/*p*)
Elbow plank	b = −0.121/*p* = 0.684	b = −1.153/***p*** = **0.02**	b = −0.369/***p*** = **0.009**
Wall sit	b = 0.013/*p* = 0.950	b = −0.417/*p* = 0.118	B = 0.075/ *p* = 0.75
Reverse plank	b = −0.589/***p*** = **0.031**	b = −0.229/*p* = 0.473	b = −0.504/*p* = 0.094
Right-side plank	b = −0.262/*p* = 0.41	b = 0.115/*p* = 0.723	b = −0.194/*p* = 0.545

Bolded *p*-values indicate statistically significant results.

## Data Availability

The original contributions presented in this study are included in the article. Further inquiries can be directed to the corresponding authors.

## Abbreviations

The following abbreviations are used in this manuscript:

OCTA	optical coherence tomography angiography
ONH	optic nerve head
IOP	intraocular pressure
VD	vessel density
ONH VD	optic nerve head vessel density
BP	blood pressure
SBP	systolic blood pressure
DBP	diastolic blood pressure
HR	heart rate
